# Perceived risk of type 2 diabetes mellitus: predictors of perceived susceptibility among young adults in Jordan

**DOI:** 10.3389/fpubh.2025.1722942

**Published:** 2026-01-14

**Authors:** Sireen Alkhaldi, Hana Taha, Mishkah BaniMustafa, Rana Al-Shimi, Jehad AlSamhori, Muhammad Alshyyab

**Affiliations:** 1 Department of Family and Community Medicine, School of Medicine, University of Jordan, Amman, Jordan; 2 School of Medicine, University of Jordan, Amman, Jordan; 3 Department of Public Health and Community Medicine, Jordan University of Science and Technology, Irbid, Jordan

**Keywords:** Jordan, perceived risk, susceptibility, type 2 diabetes mellitus, young adults

## Abstract

**Objectives:**

The growing burden of type 2 diabetes mellitus among young adults is a global public health problem. This study aimed to explore risk perception of type 2 diabetes mellitus among university students in Jordan and to identify predictors of perceived susceptibility.

**Methods:**

A cross-sectional study that used proportional stratified sampling to recruit 496 third year university students in all fields of study at the University of Jordan. Participants answered online self-administered validated Arabic questionnaire that was designed based on the constructs of the health belief model. Data was analyzed using Statistical Package for Social Sciences (SPSS) software.

**Results:**

The participants in this study evidently underestimated their risk of developing T2DM. Only 25% of students believed that they have high potential of developing T2DM in the future. Perceived susceptibility was low to moderate; it scored lowest among all dimensions of risk perception (mean = 2.86 out of 5). Results of *t*-test and ANOVA showed that perceived susceptibility was higher among students in engineering and science (*p* = 0.001), males (*p* = 0.019), with higher income (*p* = 0.008), overweight (*p* = 0.026), and students with little knowledge of T2DM (*p* = 0.027). Results of logistic regression indicated that high income level was the only significant predictor of higher perceived susceptibility (OR = 2.7. 95%CI 1.35, 3.4). Likelihood of taking preventive action was high (mean = 4.15 out of 5).

**Conclusion:**

Results of this study highlight the need for health system governance to commit to integrate national efforts to design culturally sensitive interventions to raise awareness about the risk of T2DM in Jordan, especially among young adult population.

## 1 Introduction

Type 2 diabetes mellitus (T2DM) is a global public health concern as a debilitating chronic disease. Diabetes is the leading cause of kidney failure, lower-limb amputation, and adult blindness. Its health and economic consequences have been escalating in the past decades. Globally, in 2021, around 537 million adults were living with diabetes, diabetes caused 6.7 million deaths, and caused at least 966 billion US dollars in health expenditures (1).

Three in four adults with diabetes live in low- and middle-income countries, one in three of them are undiagnosed. In Jordan, prevalence of type 2 diabetes in adults is 14.8%, resulting in 866,505 cases of diabetes (1). More children and young adults are developing T2DM recently, which can be delayed or reversed with lifestyle modifications (2).

Young adults who look healthy and active find it hard to suppose they could develop a chronic disease like diabetes, and many of them underestimate their personal risk of developing T2DM (3). Nevertheless, there is conflicting evidence regarding how young adults estimate their risk of developing T2DM (3–5). Since T2DM is a chronic metabolic disease with silent characteristics, with increasing prevalence and less awareness of the disease; health belief model (HBM) could be considered as an effective and comprehensive framework for the prevention of T2DM (6).

HBM was one of the first models developed in the 1950s, by a group of social psychologists to understand why people engage in or do not engage in disease screening and disease prevention measures (6). The HBM consists of six constructs: perceived susceptibility (feeling at risk of being exposed/ suffering from the condition), perceived severity (how serious the condition is and the related consequences of the condition), benefits of taking action toward prevention, barriers to action, self-efficacy and cues to action (7). Each of these factors are typically thought to work together additively to affect the likelihood of practicing the behavior. HBM will therefore be very appropriate to investigate perceived risk of young adults.

According to HBM, messages that effectively address perceived barriers, benefits, self-efficacy, and threats will result in the best behavior change (7). Therefore, it is expected that high susceptibility, high severity, high benefits, and low barriers will result in a high probability of taking the advised action (6). Education and training based on HBM was found effective in improving self-efficacy and implementing preventive measures such as physical activity (8).

Wide variability was observed in how studies in the literature operationalize perceived risk (9–11), WHO (12). Most of the relevant studies did not use a model or a theory as a conceptual framework for risk perception of T2DM, which limited comparability between these studies.

Literature in this field has pinpointed several variables as determinants of perceived risk of developing T2DM in young adults. University students with family history of T2DM expressed higher level of perceived risk (3, 13–17), whereas other studies found no difference related to family history (5, 16, 18). Young adult females generally revealed increased perceived risk of T2DM (15, 19–21), while other studies found no significant difference between males and females (3, 13, 16, 18). Moreover, young adults who were informed about increased diabetes risk by a physician demonstrated higher level of risk perception (3, 13). In addition, college students with higher body mass index (BMI) revealed higher perceived risk of diabetes (5, 13, 22).

In Jordan, researchers found that risk factors of T2DM are common among university students aged 18–25 especially obesity (27% had central obesity) and physical inactivity (23). Furthermore, researchers found general poor knowledge of DM among university students in Jordan (18, 24). Nevertheless, positive perception was clearly evident as 62.5% of the students agreed that weight reduction is important for DM management (24), while moderate risk perception of diabetes was reported by 2,158 young adults in South of Jordan (18). Albeik et al. (25) investigated risk perception of University of Jordan students in 2018. They concluded that students had moderate perception of susceptibility to T2DM, although they presented high perception of severity of the disease, specially appreciating its severe complications. Perceived barriers and perceived benefits were considered to be the strongest predictors of behavior as reported in previous studies in Jordan (26). Finally, researchers in Jordan concluded that health education intervention was effective in improving young adults’ attitudes toward adoption of health promotion behavior to prevent non-communicable diseases including T2DM (19). Yet, the relationship between T2DM risk perception and likelihood to take preventive actions has not been studied among young adults in Jordan.

Therefore, this study aims to use the health belief model as a framework to investigate the predictors of risk perception about type 2 diabetes mellitus among young adults in Jordan. In addition, it explored the relationship between perceived risk of developing T2DM and young adults’ likelihood to take preventive actions. The authors believe that high level of risk perception of T2DM leads to strong likelihood of taking preventive action among young adults.

## 2 Materials and methods

### 2.1 Study design and setting

This cross-sectional study investigated the perceptions about the prevention of T2DM among third-year university students. The study was conducted at the University of Jordan, the oldest and largest governmental university in Amman, the capital of Jordan, and consists of 25 schools.

### 2.2 Target population and sample

The target population of this study is third year students in all fields of study at the targeted University. We decided to include third year students because at this stage, the influence of the major field of study on their risk perception will start to appear. We excluded first and second year students because they will be studying general topics mainly, and have not all started taking classes of their major field in depth. We also excluded fourth year students because many of them will start training and internships and may not be present at the campus.

The number of students in their third year at the university in 2022 was 12,400 according to the registration unit. All 25 different schools were stratified for the purpose of this study into health/medical field (25%), engineering and science field (27%), humanities field, and business (34%) and information technology fields (14%). The researcher used proportional stratified sampling, with the number selected from each strata being proportionate to its size in the total population. Considering 95% confidence level, 5% margin of error, and *p* = 0.5 (maximum variability), calculated sample size was at least 386 participants.

Third year students who had diabetes were excluded from the study. Age of the student is irrelevant as an inclusion criteria because our focus is the influence of academic level of study. Students were invited for participation inside each stratum from different places, at different days and at different times of the day, in order to minimize selection bias. To compensate for non-response, we decided to increase sample size by 10%, in addition to 10% more for incomplete responses.

### 2.3 Study instrument

A self-administered pre-validated questionnaire was used for data collection (25). This tool included four main sections. First section is socio-demographic characteristics, including sex, field of study, parental education, and income, and family history of diabetes. In respect to health indicators, students self-reported their weight and height, smoking, commitment to healthy diet, commitment to physical activity, and family history of DM (defined as having a parent, uncle, aunt, or a grandparent with T2DM).

Second section included 13 questions about knowledge of T2DM, including causes, symptoms, treatment, and complications. Responses were categorized to yes, no, and I do not know. Each correct answer was given one point. Third section included questions related to perceptions about the prevention of T2DM. It had a total of 20 statements grouped into four main concepts adapted from the health belief model (HBM) (three statements for perceived susceptibility, six statements for perceived severity, five statements for perceived benefits and six statements for perceived barriers). Five-point Likert scale was used to measure T2DM perceptions, with a range from 1 = strongly disagree, 2 = disagree, 3 = neutral, 4 = agree, 5 = strongly agree. Some statements were back-scored (indicated in the tables). The mean for each item was calculated, in addition for the mean for each perception dimension, followed by overall mean for perception. The fourth section was intended to measure likelihood of taking recommended preventive action, with responses ranging from Strongly unlikely = 1 to strongly likely = 5 based on a five-point Likert scale.

### 2.4 Reliability

Pilot test including 45 participants was carried out, in order to determine time needed for completing the questionnaire, to check feasibility of the study and the tool and protocol, and to identify potential problems. Questionnaires included in the pilot study were included in the analysis, because only minor changes were introduced. Internal consistency of this tool was assessed using Cronbach‘s alpha for the total scale which was 0.768 and each subscale.

### 2.5 Data collection procedure

The research team collected the data during the period between March and May 2023. Data collection took place in campus; we identified places where students in each school sit and meet between classes. Research team approached students, and those who met the criteria, were asked to fill an electronic self-administered questionnaire, on the spot using a tablet provided by the research team. The number of respondents was 496 as follows: 164 (33.1%) from health/medical fields, 138 (27.8%) from engineering and science fields, 121 (24.4%) from humanities fields and 73 (14.7%) from business and IT.

### 2.6 Ethics and consent

Ethical Approval from the Institutional Review Board (IRB) at the University of Jordan was obtained (Decision no. 122/2022) dated November 27, 2022. Agreeing to participate in the study by personally responding to the questionnaire was considered an implied consent. The questionnaire started with the consent form including an item about agreement to participate. Questionnaires were anonymous, and data was stored securely at the researcher’s computer with restricted access and used only for the purpose of this research.

### 2.7 Data analysis

Responses were analyzed using the IBM Statistical Package for Social Sciences (SPSS) software (Version 23.0). Data was cleaned and checked for outliers before analysis. Simple descriptive statistics (frequencies, mean and standard deviation) were used to describe characteristics of the sample. Bivariate tests (*t*-test, Chi-Square, ANOVA) were used to verify the relationship between the variables. Multivariate analysis (logistic regression) was applied to explore predictors of perceived susceptibility to type 2 diabetes mellitus. Statistical significance was set at the *p-*value of 0.05.

## 3 Results

### 3.1 Risk perception

Table 1 presents results of the perceived T2DM severity, susceptibility, benefits, barriers, and likelihood of taking preventive action of university students in this study. Overall mean of perceived severity score is 2.98 ± 0.48, for perceived susceptibility is 2.86 ± 0.59, perceived benefits is 3.58 ± 0.54, and high likelihood of taking recommended action is 4.15 ± 0.65.

**Table 1:** Perceived T2DM severity, susceptibility, benefits, and likelihood of taking preventive action of university students in Jordan (*N* = 496).

Attitude statement	Likert scale responses^a^ (%)					Item Mean ±SD
	1	2	3	4	5	
Perceived severity						
T2DM is a serious disease	2.2	15.5	14.9	46.0	21.4	3.69 ± 1.04
T2DM cannot be prevented^b^	1.4	14.7	17.9	52.4	13.5	2.43 ± 0.94
T2DM does not scare me	7.3	38.3	23.8	26.4	4.2	2.82 ± 1.03
If I had T2DM, my academic life would be endangered	14.1	41.3	17.5	20.4	6.7	2.64 ± 1.15
If I had T2DM, it would endanger my relationships	21.2	43.3	13.9	16.3	5.2	2.41 ± 1.14
Problems I would experience from T2DM would not last long^b^	8.1	42.1	26.4	19.0	4.4	3.45 ± 1.01
Overall mean of perceived severity score (out of 5) = 2.98 ± 0.48^c^						
Perceived susceptibility						
I think my chance of getting T2DM in the future is high	11.5	35.9	27.4	20.8	4.4	2.71 ± 1.05
I do not worry about getting T2DM^b^	5.4	32.1	22.6	33.3	6.7	3.04 ± 1.06
My health status increases the chances of getting T2DM	6.5	40.7	23.2	22.6	7.1	2.83 ± 1.07
Overall mean of perceived susceptibility score (out of 5) = 2.86 ± 0.59						
Perceived benefits						
Regular exercise can prevent T2DM	0.2	3.2	8.5	59.7	28.4	4.13 ± 0.70
Weight loss cannot prevent T2DM^b^	10.7	53.2	13.1	19.6	3.4	3.48 ± 1.03
Reducing sugar consumption can prevent T2DM	0.6	5.0	12.7	65.7	15.9	3.91 ± 0.73
If I do regular screening test for blood sugar, then I would not prevent T2DM^b^	4.0	33.5	21.0	33.7	7.9	2.92 ± 1.06
Smoking cessation would prevent T2DM	3.4	13.9	24.0	49.0	9.7	3.48 ± 0.96
Overall mean of perceived benefits score (out of 5) = 3.58 ± 0.54						
Likelihood of taking recommendation						
To follow physicians’ orders related to my health	0.6	2.2	8.9	45.8	42.5	4.27 ± 0.76
To have healthy diet and avoid too much sugar and fat	1.4	4.4	10.7	45.4	38.1	4.14 ± 0.87
To have healthy weight	2.0	5.8	9.1	46.2	36.9	4.10 ± 0.93
To stop smoking	3.8	6.0	12.9	27.0	50.2	4.14 ± 1.09
To do regular exercise for 30 min for five times weekly	2.0	4.2	10.5	48.2	35.1	4.10 ± 0.89
Overall mean of likelihood of taking recommendation score (out of 5) = 4.15 ± 0.65						

a 1 = Strongly disagree, 2 = Disagree, 3 = Neutral, 4 = Agree, 5 = Strongly Agree.

b Statement is reverse-scored.

c Overall mean of each perception category.

### 3.2 Sample characteristics and bivariate analysis

This section presents main results of this study. The total number of students was 496, with the majority of the sample being females (71.6%). The sample is distributed proportionately over different specialties in the universities. More than half of students come from families with monthly income of 7,500 JDs or less. More than 26% of students are smokers, and more than 40% of them are overweight or obese. The majority of students (93%) reported family history of diabetes, where one or more of parents, uncles, aunts or grand parents had diabetes. Only 55% of students are usually or always committed to healthy diet and around 44% of then have regular physical activity (see Table 2).

**Table 2:** Socio-demographic characteristics and relationship between perceptions^a^ of severity, susceptibility and benefits scores with socio-demographic and health-related variables, results of *t*-test and ANOVA (*N* = 496).

Variable	Categories	%	Perceived severity		Perceived susceptibility		Perceived benefits	
			Mean	*p*-value	Mean	*p-*value	Mean	*p-* value
Gender	Male	18.4	3.02 ± 0.51	0.193	2.96 ± 0.62	**0.019**	3.49 ± 0.60	**0.011**
	Female	71.6	2.96 ± 0.46		2.81 ± 0.58		3.62 ± 0.51	
Field of study	Engineering and Science	27.8	3.06 ± 0.54	0.123	3.03 ± 0.64	**0.001**	3.06 ± 0.55	**0.048**
	Health/Medical	33.1	2.96 ± 0.49		2.81 ± 0.63		3.25 ± 0.48	
	Humanities	24.4	2.95 ± 0.41		2.77 ± 0.45		3.02 ± 0.52	
	Business and IT	14.7	2.92 ± 0.42		2.79 ± 0.56		3.10 ± 0.55	
Monthly income (*n* = 179)	<500 JDs	19.5	2.98 ± 0.39	0.529	2.81 ± 0.55	**0.008**	3.58 ± 0.58	0.298
	500–750 JDs	37.9	2.95 ± 0.44		2.78 ± 0.50		3.64 ± 0.54	
	750–1,200 JDs	18.8	3.04 ± 0.49		2.89 ± 0.59		3.52 ± 0.51	
	>1,200 JDs	23.8	2.98 ± 0.58		3.01 ± 0.73		3.58 ± 0.55	
Smoking	Yes	26.2	2.99 ± 0.47	0.825	2.94 ± 0.62	0.072	3.45 ± 0.60	**0.001**
	No	73.8	2.98 ± 0.48		2.83 ± 0.58		3.63 ± 0.51	
BMI	Underweight	4.6	2.88 ± 0.39	0.108	2.62 ± 0.54	**0.026**	3.49 ± 0.57	0.369
	Normal weight	53.2	3.01 ± 0.52		2.81 ± 0.63		3.61 ± 0.53	
	Overweight	27.2	2.99 ± 0.44		2.95 ± 0.55		3.60 ± 0.51	
	Obese	15.0	2.87 ± 0.39		2.91 ± 0.53		3.50 ± 0.63	
Family history of DM	Yes	93.1	2.99 ± 0.49	0.223	2.87 ± 0.58	0.08	3.59 ± 0.54	0.231
	No	6.9	2.88 ± 0.33		2.69 ± 0.75		3.48 ± 0.54	
Knowledge of T2DM^b^	Good	53.3	2.96 ± 0.40	0.063	2.8 ± 0.51	**0.027**	3.75 ± 0.53	**0.001**
	Deficient	46.7	3.01 ± 0.42		2.92 ± 0.67		3.40 ± 0.53	
Healthy diet	Never or rarely	15.5	2.96 ± 0.42	0.349	2.89 ± 0.62	0.639	3.43 ± 0.58	**0.001**
	Sometimes	18.6	2.98 ± 0.52		2.85 ± 0.62		3.54 ± 0.51	
	Usually	38.3	2.95 ± 0.46		2.88 ± 0.57		3.60 ± 0.52	
	Always	17.6	3.06 ± 0.48		2.79 ± 0.57		3.75 ± 0.56	
Physical activity	Never or rarely	17.0	2.92 ± 0.41	0.419	2.86 ± 0.60	0.368	3.49 ± 0.51	**0.015**
	Sometimes	29.2	2.99 ± 0.52		2.89 ± 0.63		3.61 ± 0.54	
	Usually	26.6	3.00 ± 0.52		2.88 ± 0.58		3.56 ± 0.51	
	Always	17.1	3.01 ± 0.43		2.80 ± 0.54		3.73 ± 0.62	

a 1 = Strongly disagree, 2 = Disagree, 3 = Neutral, 4 = Agree, 5 = Strongly Agree.

b Knowledge was dichotomized as follows: 0–10 = deficient knowledge, > = 11 is good knowledge.

Bold means value is statistically significant.

### 3.3 Bivariate analyses

In Table 2, relationships between mean perceptions of severity, susceptibility and benefits scores and socio-demographic and health-related variables are presented. Perceived susceptibility is significantly higher among males (*p* = 0.019), students studying engineering and science (*p* = 0.001), those with highest monthly income (*p* = 0.008), and students who are overweight (*p* = 0.026). Regarding perceived benefits, non-smokers, students committed to healthy diet and those committed to physical activity showed significantly higher perceived benefits (*p* = 001, *p* = 0.001 and *p* = 0.015), respectively.

### 3.4 Regression analyses

Table 3 presents result of logistic regression carried out to assess the predictors of perceived susceptibility to T2DM. The overall model was statistically significant when compared to the null model, (*χ*^2^ (11) = 31.68, *p* = 0.001). This model explained 9.7% (Nagelkerke *R*^2^) of the variance in perceived susceptibility to T2DM. This indicates weak relationship between independent variables and the dependent variable. This clearly implies the presence of other variables not included this this study, which may predict perceived susceptibility. Logistic regression results indicated that, holding other variables constant, students with highest family income (>1,200 JD’s) had 2.7 times the odds of perceived susceptibility to T2DM compared to students with lowest income level (*p* = 0.005, 95%CI = 1.35–3.40). Furthermore, and surprisingly, holding other variables constant, the odds of perceived susceptibility to T2DM was 45% lower among students in health sciences, compared to students in engineering and science fields (*p* = 0.025, 95%CI = 0.31–0.93).

**Table 3:** Predictors of perceived susceptibility^a^, binary logistic regression results (*N* = 496).

Variable	Categories	*p*-value	OR	95%CI
Monthly family income (*n* = 179)	<500 JD (reference)			
	500–750 JD	0.520	0.82	0.44–1.50
	750–1,200 JD	0.312	1.45	0.70–2.98
	>1,200 JD	**0.007**	**2.70**	**1.35–3.40**
Sex	Male (reference)			
	Female	0.269	1.30	0.82–2.06
Field of study	Engineering and Science (reference)			
	Health/Medical	0.054	0.50	0.31–1.02
	Humanities	0.355	0.82	0.39–1.70
	Business and IT	0.270	0.88	0.38–1.36
Family history of T2DM	No (reference)			
	Yes	0.77	1.13	0.49–2.62
BMI	Normal weight (reference)			
	Underweight	0.167	0.42	0.12–1.44
	Overweight	0.077	0.58	0.31–1.06
	Obese	1.000	1.00	0.53–1.90
Knowledge of T2DM	Good (reference)			
	Deficient	0.149	0.73	0.47–1.12

a Perceived susceptibility was dichotomized into: 0–9 is low susceptibility and > = 10 is high susceptibility (total perceived susceptibility score = 15). Bold means value is statistically significant.

## 4 Discussion

According to the health belief model, a person’s risk perception (perceived susceptibility and perceived severity) of developing T2DM is a critical factor that determines adopting healthy lifestyles and adopting preventive interventions to reduce this risk. This study examined risk perception of type 2 diabetes mellitus among third year university students in Jordan. It aimed to indicate predictors of perceived susceptibility to T2DM including sex, family income, field of study, family history of T2DM and BMI.

### 4.1 Perceived severity and susceptibility

Our results highlighted that third year university students considerably underestimated their risk of developing T2DM despite the fact that risk factors are well known. Only 25% of students believed that they have high potential of developing T2DM in the future. Perceived susceptibility was low to moderate; it scored lowest among all dimensions of risk perception (mean = 2.86 out of 5). This finding supports similar results reported in Jordan (18, 25), in USA (5, 13, 20), in Germany (27), and in Uganda (28). This may be explained by the low perceived severity of T2DM among these students. Despite the low to moderate perceived susceptibility, students revealed high intention to take recommended preventive action.

Results revealed lower than expected perceived severity (mean = 2.98); 67.5% of students believed that T2DM is a serious disease. These young adults seem to have low appreciation of how debilitating T2DM can be as a chronic disease. Therefore, university setting can provide a valuable chance to reach adults at this specific stage of life to spread T2DM risk reduction messages.

### 4.2 Predictors of T2DM perceived susceptibility

Results revealed monthly income (the most prominent socioeconomic status indicator) as the only variable that remained as a significant predictor of perceived susceptibility to T2DM using multivariate analysis. The concerns of people with high socioeconomic status about T2DM may be related to higher exposure to health information, shaped by messages and media (10). Reviewing the literature in this area, we did not find any study that directly investigated the influence of family income level on risk perception. However, many studies found that low income is highly connected with developing type 2 diabetes (29), this may reflect that families with low income level have lower levels of perceived risk of T2DM.

This study showed that males perceived susceptibility to T2DM significantly higher compared to females, which contradicted our expectation. This comes in congruence with previous results among university students in Jordan (18, 25), in USA (13), and in Germany (27). On the contrary, most of the literature in this area reported females to express higher levels of perceived risk to T2DM (10, 15, 19–21). Nevertheless, after controlling for other variables using logistic regression, this difference between males and females disappeared.

Surprisingly, medical students had lower perceived susceptibility compared to engineering and science students even at the third year of study, although this difference disappeared in multivariate analysis. This contradicts previous results from in Jordan (18, 25), where medical and health students expressed highest level of perceived risk to T2DM compared to all other specialties. In general, the difference in perceived susceptibility according to the field of study of college students, was not explored in most of the studies in the literature.

We expected in this study to find medical and health students possessing the highest risk perception of T2DM, due to their extensive exposure to basic medical sciences. This can be understood when results explained that knowledge of T2DM was not proven to be a predictor of perceived susceptibility. Here, it seems that students with better knowledge of T2DM do feel more confident of their ability to control their risk of developing T2DM in the future. Nevertheless, only perceived benefit of preventive measures was highest among medical and health science students.

The findings of this study demonstrated difference in the perceived susceptibility to T2DM in relation to BMI of the students. Students who are overweight had higher perceived susceptibility, though not significant in multivariate analysis. Same results were consistently reported in Jordan and other countries (5, 13, 22, 25, 27). This can be related to their knowledge about the negative health effects of excess body weight, including T2DM.

Family history is a well-documented risk factor of T2DM. In this study; students who had family history of T2DM expressed the same perceived susceptibility of getting T2DM compared to those with no family history. Although 93% of students had family history of T2DM, only 25% of students in this study believe that their chance of getting T2DM in the future is high. This is consistent with results of other studies in the literature (5, 16, 18). In this line, previous research found that knowledge about hereditary genetic predisposition to diabetes did not increase motivation to change lifestyle to prevent T2DM (30). This can be explained by the idea that knowledge of the genetic cause of T2DM, may lead to the belief that prevention is beyond our control, and that preventive measures will have no real benefit. Nevertheless, a number of other studies reported family history to be significantly associated with higher risk perception (3, 13, 14, 27). Since family history is not modifiable, young adults can benefit most from interventions to increase knowledge about the high risk associated with their family history, where lifestyle changes and early health interventions can be introduced to lower their future risk. University life and environment can be a precious opportunity for promotion of physical activity and healthy diet among these students.

### 4.3 Limitations

The findings of this study should be interpreted carefully because of some limitations. First, this study included students enrolled in the University of Jordan at their third academic year, which limits generalizability to students in other levels of study. However, due to the similarity of the Jordanian people’s political, social, and economic status, the findings might be generalized and can somehow reflect the perception among various Jordanian university students from different levels. Another limitation was the convenient selection of participants in each strata, which indicates potential selection bias to the study. Although random sampling is the ideal way to select participants in each strata, that was impractical for the purpose of this study.

Information bias may have been introduced to the study because data were collected depending on self-administrated questionnaires. This applies to information about income, height and weight, which were self-reported by participating students and was applied for practical reasons. This may have led to under-estimation of obesity in this study. Furthermore, this study has limitation in its predictive ability of the model used, due to the low variance with modest Nagelkerk *R*^2^ reported. This suggests that other social or cultural variable, not addressed in this study, may have influenced participants’ responses.

### 4.4 Implications

Results of this study could inform health-related strategic efforts in Jordan concerning T2DM, by highlighting the essential need to increase awareness of diabetes risk. Our findings provide rationale for planning and designing appropriate interventions to educate university students about their real life-long risk of developing T2DM, with the modifiable lifestyle risk factors. Students in third year of study are at an important developmental stage of their life, bearing the responsibility for making choices for their personal health and establishing healthy behaviors (19, 31). It is also instrumental to address the importance of risk communication between health professionals and patients on the primary care level in Jordan.

## 5 Conclusion

Students in Jordan reported moderate to low levels of perceived susceptibility to T2DM and severity of T2DM, but high likelihood of taking recommended action. High income was the only variable showing significant association. Findings have shed light on the essential need for national multi-sectoral efforts in Jordan to raise knowledge related to the risk of T2DM at all population levels. Future research is needed regarding effective interventions to raise risk perception of major chronic diseases in Jordan including T2DM.

## Data Availability

The original contributions presented in the study are included in the article/Supplementary material, further inquiries can be directed to the corresponding author.
